# Editorial for the Special Issue on “Multidisciplinary Insights on Bone Healing (Volume II)”

**DOI:** 10.3390/biology14010032

**Published:** 2025-01-03

**Authors:** Alexandre Anesi, Mattia Di Bartolomeo

**Affiliations:** 1 Department of Medical and Surgical Sciences for Children and Adults, Cranio-Maxillo-Facial Surgery, University of Modena and Reggio Emilia, Largo del Pozzo 71, 41125 Modena, Italy; 2 Department of Oral and Maxillofacial Sciences, Sapienza University of Rome, 00161 Rome, Italy; mattiadiba@hotmail.it

As the population ages and differences among sexes and age groups become more pronounced, the research on bone healing and damage mechanisms continues to advance, with evaluation conducted in both pre-clinical and clinical settings [1,2]. The analysis of the results from various clinical scenarios is essential and should be performed in a multi-disciplinary manner, taking into account underlying pathogenetic mechanisms, radiographic evaluations, and macroscopic clinical results [3,4,5]. New study models should be implemented, and existing ones re-discovered, while also challenging the status quo to explore new therapeutic possibilities [6,7]. Attention is also particularly focused on new technologies that can have multiple impacts on all aspects of bone regenerative processes. New osteotomes, such as the piezosurgical ones, have been developed to reduce the amount of bone damage during surgical procedures [8]. New technologies have also been developed in order to enhance bone healing processes, such as pulsed electro-magnetic fields [9]. The use of artificial intelligence tools is on the rise, for example, to automatically segment the anatomical structures involved in these processes, thereby facilitating the evaluation of results [10]. Finally, new biomaterials are continuously developed and improved, both in terms of cost-effectiveness and final outcomes, expanding the arsenal of regenerative tools and scaffolds available for clinical use [4,11,12,13,14,15]. All of these topics have been encountered in this Special Issue, entitled “Multidisciplinary Insights on Bone Healing (Volume II)”: some of them were also discussed in the first volume and they were expanded upon here, while new ones have been introduced in this edition. A total of 11 papers have been published, covering a wide range of topics:

- A thorough narrative review on bone disorders of children affected by chronic kidney disease, starting from the previous term as “renal osteodystrophy” and highlighting the current, correct definition of “chronic kidney disease–mineral and bone disorder (CKD-MBD)” and all of its features, from pathogenesis to treatment [2];
- An experimental study on the osteoconductive and biocompatibility properties of Poly(ε-caprolactone) (PCL) combined with 20% Tricalcium Phosphate (TCP), following a 3D-printing procedure and a sterilization process, aimed at improving the cost-effectiveness of jawbone augmentations in oral surgery [14];
- A comprehensive review regarding the role of sex differences in bone health and healing, highlighting the differences present in the literature and the need for a more thorough evaluation of the key factors that may impact bone healing processes [1];
- A research study on the capacity of magnesium to induce osteoclast differentiation and how the presence of zoledronate enhances its effectiveness. The use of zoledronate places the patient at risk of developing bisphosphonate-related osteonecrosis of the jaws, and this study opens new scenarios on the role of magnesium as a topical therapy for this condition [7];
- A review of the use of the chorioallantoic membrane (CAM) model as a preclinical tool to experimentally evaluate the potential for bone regeneration of functionalized 3D constructs, particularly in those critical size defects that do not heal spontaneously [6];
- A research study on the interaction between bone marrow-derived multipotent mesenchymal cells and 3D-printed substrates: poly(e-caprolactone) combined with 20% tricalcium phosphate (PCL + 20% β-TCP) and L-polylactic acid (PLLA) combined with 10% hydroxyapatite (PLLA + 10% HA). This paper focuses on the homing and differentiation processes of these cells and how they interact with these 3D-printed scaffolds [14];
- A pre-clinical study focused on periosteal osteochondral ossification and the role of Fibroblast Growth Factor Receptor 3 (FGFR3), specifically uncovering the role of microRNAs and their interaction networks both in vitro and in vivo models [16];
- A clinical study on the relationship between alkaline phosphatase levels and radiographic features of tibial fractures (evaluated using the Radiographic Union Scale for Tibial Fractures), demonstrating their complementarity in the evaluation of bone consolidation [5];
- An updated review focused on osteo-immunologic processes in rheumatoid arthritis and spondylarthritis, highlighting the pathogenetic mechanisms of bone erosion and the systemic osseous involvement, which ultimately lead to systemic bone loss, osteoporosis, and increased skeletal fragility in these two conditions [3];
- A retrospective study on neurosurgical and maxillo-facial patients investigating the role of polyetheretherketone (PEEK) implants in bone regeneration in a clinical setting, emphasizing the importance of patient-specific factors and implant design to obtain relevant results [4];
- A comprehensive review of the combination of bone tissue engineering and nanotechnology, highlighting its role in new regenerative strategies and its advantages, taking into account the results of the use of nanoparticles both in vitro and in vivo studies [13].
